# Interaction of 5-[4′-(*N*-Methyl-1,3-benzimidazol-2-yl)phenyl]-10,15,20-tri-(*N*-methyl-3′-pyridyl)porphyrin Triiodide with SARS-CoV-2 Spike Protein

**DOI:** 10.1134/S1070363222060123

**Published:** 2022-06-20

**Authors:** S. A. Syrbu, A. N. Kiselev, M. A. Lebedev, Yu. A. Gubarev, E. S. Yurina, N. Sh. Lebedeva

**Affiliations:** 1 G.A. Krestov Institute of Solution Chemistry of the Russian Academy of Sciences, 153045 Ivanovo, Russia; 2 Ivanovo State University of Chemistry and Technology, 153000 Ivanovo, Russia

**Keywords:** porphyrins, spectroscopy, spike protein, SARS-CoV-2 virus, inhibition, inactivation

## Abstract

The results of experimental studies of the interaction of the S-protein with a monohetaryl-substituted porphyrin containing a benzimidazole residue are presented. It has been revealed that the S-protein forms high-affinity complexes with the specified porphyrin. The porphyrin binding by the SARS-CoV-2 S-protein has proceeded stepwise; at the first stage, the driving force of the complexation is electrostatic interaction between the surface negatively charged regions of the protein and cationic substituents of the porphyrin. At the second stage, the target complex of the S-protein with the porphyrin is formed. It has been established that the introduction of 5-[4′-(*N*-methyl-1,3-benzimidazol-2-yl)phenyl]-10,15,20-tri-(*N*-methyl-3′-pyridyl)porphyrin triiodide into a solution of the S-protein complex with the angiotensin-converting enzyme leads to the replacement of the latter with the porphyrin. Displacement of the angiotensin-converting enzyme from the complex with the S-protein under the action of 5-[4′-(*N*-methyl-1,3-benzimidazol-2-yl)phenyl]-10,15,20-tri-(*N*-methyl-3′-pyridyl)porphyrin triiodide is the experimental evidence for the porphyrin binding at the receptor-binding domain of the S-protein.

New coronavirus SARS-CoV-2 causing COVID-19 has significantly impacted human health worldwide. Over the recent few months, variants of SARS-CoV-2 with new mutations of the S-protein have appeared, affecting the epidemiological and clinical aspects of the COVID-19 pandemic [1–3]. Different variants of the virus mutation can accelerate its transmission and/or increase the risk of reinfection and reduce the protection ensured by neutralizing monoclonal antibodies and vaccination [4]. The spike protein of the SARS-CoV-2 provides for the binding with the angiotensin-converting enzyme located at the host cells surface (АСЕ2) and for the fusion of the membranes of the virus and the cell.

The spike protein also induces the reaction of the neutralizing antibodies and is thus among the main targets in the development of drugs and vaccines [5, 6]. The spike protein exserted at the surface of the virion consists of three identical chains of the S-protein. Each chain consists of two subunits: S1 and S2. The S1 subunit is responsible for the binding with the receptor, while S2 is involved in the fusion of the virus and the host cell membranes [7]. The S1 subunit contains the N-terminal domain (NTD) and the receptor-binding domain (RBD), the latter being complementary to the АСЕ2 receptor of the host cell. NTD is the outmost domain, relatively more open; it is located from three sides thus imparting triangular shape to the protein. The cryogenic electron microscopy studies have revealed [8] certain flexibility of the NTD and RBD, but the conformation dynamics has been mainly between the closed and the open conformations. The interaction between RBD and АСЕ2 induces strong conformation change in the S2 subunit, followed by splitting of the S-protein into the S1 and S2 subunits under the action of the host enzymes (furin and transmembrane serine protease), which finally leads to fusion of the virus shell with the cellular membrane of the host and release of the viral nucleus into the cell cytoplasm [9–12].

Evidently, a drug binding with the S-protein can inhibit the virus penetration in the cells, due to the competition with the binding with the АСЕ2, or prevent the conformation changes in the S-protein required for the membranes fusion. The procedure for the synthesis of 5-[4′-(*N*-methyl-1,3-benzimidazol-2-yl)phenyl]-10,15,20-tri-(*N*-methyl-3′-pyridyl)porphyrin triiodide (N-por) developed via molecular docking with the S-protein of SARS-CoV-2 having revealed the highest binding affinity, has been elaborated earlier in [13]. In this study, we aimed to experimentally investigate the processes of the complex formation of the monohetaryl-substituted porphyrin baering a benzimidazole moiety (Scheme 1) with the S-protein and the complex of the latter with АСЕ2.

**Scheme:**
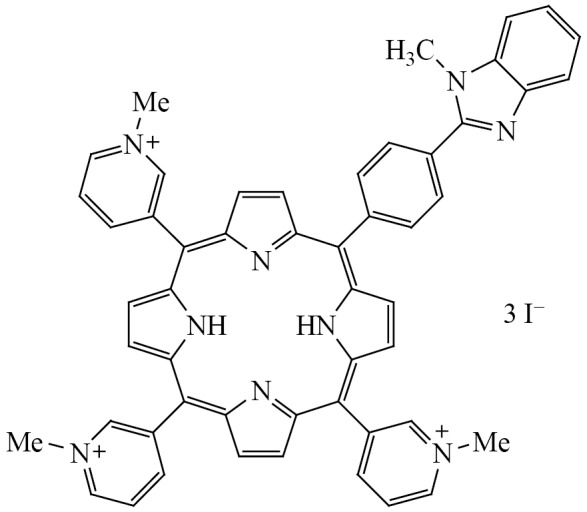
***1.***

It should be noted that the porphyrin binding occurs at the surface of the RBD of each chain in the S-protein, as per the molecular docking data. The porphyrin compound is located between the RBD regions of two neighbor chains, simultaneously interacting with both amino acid residues of the RBD (Fig. 1) [13].

**Fig. 1.**
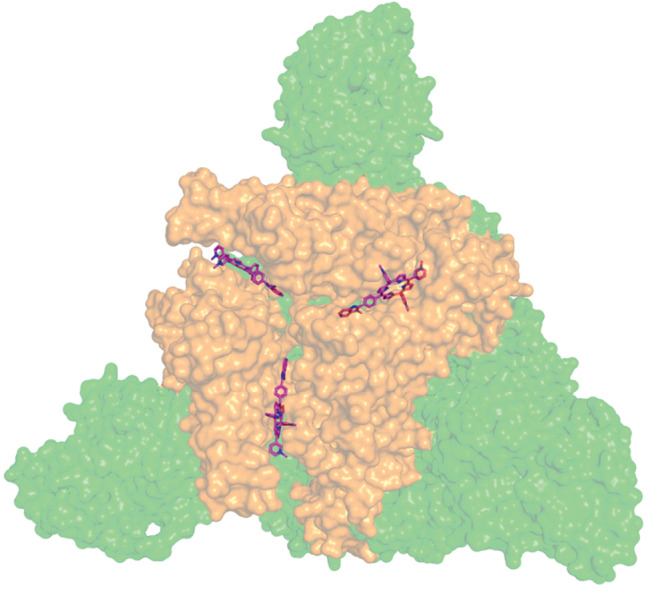
Results of molecular docking of N-por with the S-protein. The receptor-binding domains is shown in orange.

The S-protein is a large coronavirus protein, its molecular mass being of 150 kDa; the protein surface bears relatively many hydrophobic (uncharged) fragments of the polypeptide chain [14, 15]. Therefore, concentrating of the protein required for titration of a porphyrin compound solution with it is undesirable. High concentration of the protein can induce its self-association. Titration of the porphyrin with a concentrated protein solution would be accompanied by dilution of the latter, leading to the shift of the slowly establishing aggregation equilibriums. That effect would distort the results of the porphyrin titration with the protein. However, the spectral manifestation of the porphyrin interaction with the protein could be thus correctly estimated. Therefore, at the first stage we performed titration of the porphyrin solution with the S-protein.

Typical changes in the electronic absorption spectrum of the porphyrin are shown in Fig. 2. It is to be seen that the initial titration steps (up to the protein : porphyrin molar ratio 1 : 1.6, Fig. 2a) was accompanied by the increase in the absorbance in the region of the Soret band of the porphyrin. We attributed those changes to fast surface electrostatic interaction of the cationic porphyrin with the negatively charged regions of the protein surface. Further titration was accompanied by the splitting of the Soret band: an absorption band with the bathochromic shift of 2–3 nm appeared in the spectrum (Fig. 2b). That phenomenon evidenced the change in polarization of the π-system of the porphyrin due to the change in its solvate surrounding. Likely, the changes documented in Fig. 2b were related to the formation of the target complex of the S-protein with the monohetaryl-substituted porphyrin. That suggestion was indirectly confirmed by a similar bathochromic shift of the porphyrin Soret band upon the change of the aqueous solvent to DMF (Table 1).

**Fig. 2.**
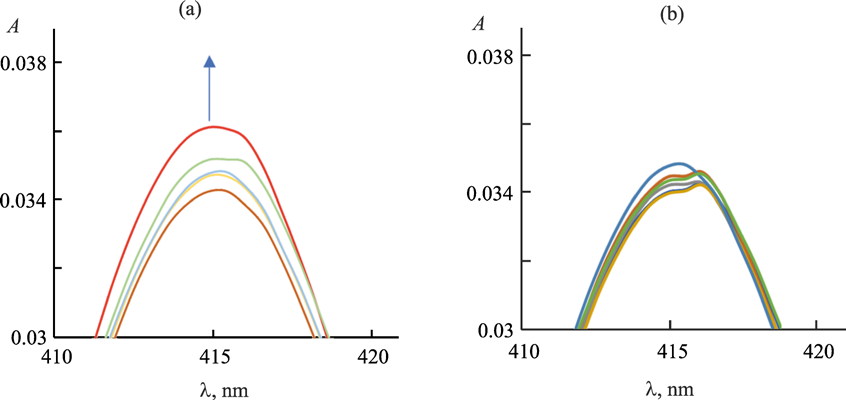
Changes in the Soret band range during titration of N-por (2×10^–7^ mol/L) with the S-protein (3.98×10^–6^ mol/L) in PBS. The titration was performed until the protein : porphyrin molar range 1 : 1.6 (a) and 1 : 0.75 (b).

**Table 1.** Spectral parameters of N-por in DMF and PBS

λ, nm	Peak area	FWHM		λ, nm	Peak area	FWHM
DMF				PBS		
419.6	38.3	17.5		414.3	29.2	19.3
512.6	9.4	48.3		513.7	2.1	26.9
546.9	4.6	26.7		535.8	0.6	26.7
586.8	7.6	45.6		578.7	0.8	27.5
639.7	4.9	29.8		636.3	0.3	29.8

Quantitative parameters reflecting the affinity of the S-protein to the considered porphyrin were obtained via spectrophotometric titration of the S-protein (Table 2). Concentration of the S-protein in the solution did not exceed 1.67×10^–6^ mol/L. The increase in the porphyrin concentration at each titration step was accounted for by subtraction of the spectrum of the porphyrin solution in the pure solvent with the same concentration.

**Table 2.** Parameters of the S-protein affinity to N-por

System	Affinity		Number of binding sites	
	electronic absorption spectroscopy	fluorescence spectroscopy	electronic absorption spectroscopy	fluorescence spectroscopy
S-protein–N-por	2.4×10^6^	5.94×10^5^	1.7	3.6
	1.9×10^5^	1.18×10^5^	2.6	4.7
S-protein–ACE2–N-por	2.23×10^5^	1.56×10^5^	0.9	2.2
	2.73×10^5^	1.25×10^5^	1.7	2.4

Fluorescence spectroscopy was applied to analyze the porphyrin–protein interactions via monitoring of the change in the emission intensity. The fluorescence of the monohetaryl-substituted porphyrin in the complex with the S-protein was stronger than that in the pure solvent (Fig. 3). That fact could be due to the displacement of the fluorescence quenchers (iodide ions) in the outer coordination sphere upon the porphyrin binding at the RBD of the protein or the shift of the association equilibriums involving the protein.

**Fig. 3.**
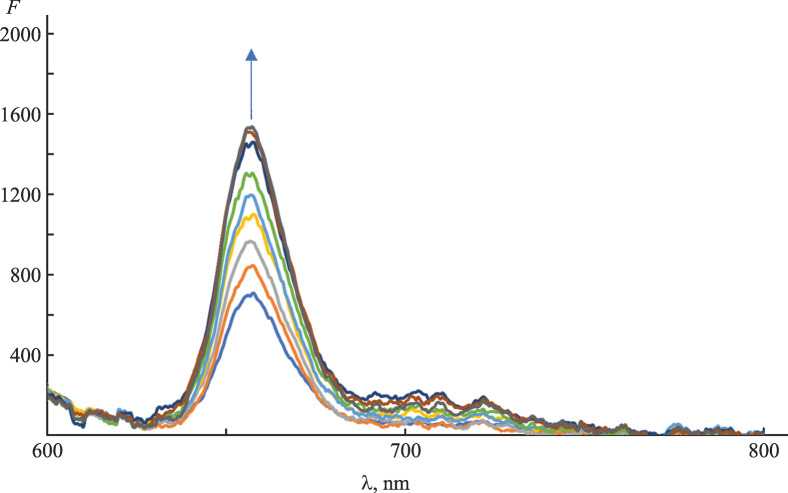
Difference fluorescence spectra of N-por in a solution of the S-protein in PBS.

The latter reason was more probable, since direct as well as reverse titration were accompanied by weakening of the light scattering observed during registration of the fluorescence spectra excited at λ_ex_ 425 nm. The associative interactions involving porphyrins were labile and therefore did not contribute significant inaccuracy into the calculated affinity parameters (Table 2).

The next issue considered in the study was assessment of the possibility of the ACE2 displacement with the porphyrin from the complex with the S-protein. The S-protein complex with the ACE2 was prepared via the components mixing in the buffered solution. Electronic absorption spectrum of the complex was poorly informative; the addition of the ACE2 to the corresponding solution led to the increase in the absorbance at about 282 nm due to the presence of the ACE2, on top of strong absorption band of the S-protein with the maximum at 237 nm. On the contrary, fluorescence spectra of the S-protein complex with the ACE2 significantly differed from those of the individual substances. The complex revealed a broad fluorescence band with the maximum at 338 nm. The studied solution of the S-protein complex with the ACE2 was optically transparent, and the reflection spectra at the excitation wavelength (295 nm) were typical of the non-aggregated protein solutions.

Titration of the S-protein–ACE2 complex with the N-por led to significant changes in the solution. Strengthening of the reflection spectrum was observed already at the early stages of the substitution titration, related to the ACE2 displacement from its complex with the S-protein. The Scatchard parameters reflecting the affinity of the N-por to the S-protein–ACE2 complex are listed in Table 2. Similarly to the case of the S-protein binding with the N-por, the plot was nonlinear in the Scatchard coordinates, rather showing two parts evidencing the formation of two types of the complexes in the system. It was reasonable to assume that, as in the case of the N-por–S-protein complex formation, the primary complex was due to electrostatic interaction. The affinity of the N-por to the S-protein–ACE2 complex was almost an order of magnitude lower than that towards the S-protein, which was likely explained by the required substitution/displacement of the АСЕ2. Stability of the N-por–S-protein complex obtained upon the ACE2 displacement was about twice higher than the analogous parameter obtained for the N-por complex with the individual S-protein. Those facts evidenced the preferable reorganization of the N-por binding site in the S-protein occurring via decomposition of the S-protein complex with the ACE2. Hence, the results of the substitution titration clearly demonstrated that the introduction of the N-por into the solution of the S-protein–ACE2 complex led to the decomposition of the S-protein complex with the angiotensin-converting enzyme. The competing of the N-por and ACE2 for the binding with the S-protein confirmed that the porphyrin binding occurred at the RBD site of the S-protein.

In summary, the performed spectral study of the S-protein interaction with the monohetaryl-substituted porphyrin bearing a benzimidazole residue confirmed that the S-protein formed high-affinity complexes with the considered porphyrin. Binding of the porphyrin with the S-protein occurred stepwise; the driving force at the first step being the electrostatic interaction between the surface negatively charged regions of the protein and the cationic substituents of the porphyrin. That stage was reflected in the porphyrin solution spectra as the strengthening of the Soret band. The second stage consisted in the complex formation between the porphyrin and the RBD of the S-protein (the target complex). Formation of that complex was manifested in the spectra as the decrease in the absorbance and bathochromic shift of the Soret band as well as strengthening of the porphyrin fluorescence.

The results of the substitution titration of the S-protein–ACE2 complex with N-por clearly demonstrated that the N-por induced decomposition of the S-protein complex with the angiotensin-converting enzyme. Competition of the N-por and ACE2 for the binding with the S-protein confirmed that the porphyrin interacted with the RBD site of the S-protein.

## EXPERIMENTAL

Synthesis and spectral parameters of water-soluble 5-[4′-(*N*-methyl-1,3-benzimidazol-2-yl)phenyl]-10,15,20-tri-(*N*-methyl-3′-pyridyl)porphyrin triiodide have been presented in Ref. [16]. The S-protein of SARS-CoV-2 was expressed, isolated, and purified at the Lobachevsky State University of Nizhny Novgorod. Molecular mass and authenticity of the target protein were confirmed by means of electrophoresis in polyacrylamide gel and immunoblotting. The experiments were performed in the phosphate buffered solution with рН 7.4 (PBS, Sigma).

Electronic absorption spectra and fluorescence spectra were recoded using an AvaSpec-2048 spectrophotometer (AvantesBV, the Netherlands) at 25°C in a constant-temperature cell. A monochromatic light diode UVTOP-295 (SensorElectronicTechnology, Inc. USA) was used as the exciting light source during the fluorescence studies.
